# Trust-Based Decision-Making in the Health Context Discriminates Biological Risk Profiles in Type 1 Diabetes

**DOI:** 10.3390/jpm12081236

**Published:** 2022-07-28

**Authors:** Helena Jorge, Isabel C. Duarte, Carla Baptista, Ana Paula Relvas, Miguel Castelo-Branco

**Affiliations:** 1 Coimbra Institute for Biomedical Imaging and Translational Research, CIBIT/ICNAS, Faculty of Medicine, University of Coimbra, 3004-531 Coimbra, Portugal; helena.vmjorge@gmail.com (H.J.); catarinaduarte@uc.pt (I.C.D.); 2 PIDFIF-Inter-University PhD Program in Clinical Psychology, Family Psychology and Family Intervention, Faculty of Psychology and Educational Sciences of Coimbra, Faculty of Psychology of Lisbon, 1649-013 Lisboa, Portugal; 3 Endocrinology, Diabetes and Metabolism Department (SEMD), University and Hospital Center of Coimbra, 3004-561 Coimbra, Portugal; cfmbaptista@gmail.com; 4 Faculty of Psychology and Educational Sciences & Center for Social Studies, University of Coimbra, 3004-531 Coimbra, Portugal; aprelvas@fpce.uc.pt

**Keywords:** human decision-making, diabetes type 1, context-dependent trust game, probabilistic learning, norm violation, treatment adherence, metabolic control

## Abstract

Theoretical accounts on social decision-making under uncertainty postulate that individual risk preferences are context dependent. Generalization of models of decision-making to dyadic interactions in the personal health context remain to be experimentally addressed. In economic utility-based models, interactive behavioral games provide a framework to investigate probabilistic learning of sequential reinforcement. Here, we model an economic trust game in the context of a chronic disease (Diabetes Type 1) which involves iterated daily decisions in complex social contexts. Ninety-one patients performed experimental trust games in both economic and health settings and were characterized by a multiple self-report set of questionnaires. We found that although our groups can correctly infer pay-off contingencies, they behave differently because patients with a biological profile of preserved glycemic control show adaptive choice behavior both in economic and health domains. On the other hand, patients with a biological profile of loss of glycemic control presented a contrasting behavior, showing non-adaptive choices on both contexts. These results provide a direct translation from neuroeconomics to decision-making in the health domain and biological risk profiles, in a behavioral setting that requires difficult and self-consequential decisions with health impact. Our findings also provide a contextual generalization of mechanisms underlying individual decision-making under uncertainty.

## 1. Introduction

A large array of behavioral studies investigating decision-making under uncertainty have been used to explain individual preference differences through experimental neuroeconomic games involving a difficult choice under ambiguity with money as reward [1,2,3,4,5]. These studies involve the following cognitive processes: option representation, valuation, action selection, outcome evaluation and learning, using update rules.

Most studies focus on the valuation system, but only in the monetary domain. It remains to be investigated how people assign a value to potential health rewards and punishments that could result from the choice. Previous studies have been based on subjective monetary value; sensitivity to reward and how delayed a reward will be in time and consider the factors that may contribute to this computation [6,7,8]. These include payoff, probability, variance, cost/effort and context. It is also important how individual perceptions about outcome probability relate to real outcome [4]. In this context, estimation, anticipation risk, error monitoring and prediction error are important factors. It is also important to consider sequential decision-making as risk behavior changes due to probabilistic learning, including the balance between previous choices and experienced outcomes [9,10,11]. Finally, it is important to investigate prosocial contexts/situations by keeping some contingencies, relevant to social interactions [12], in particular in the health domain.

Here we address decision-making in the context of self-consequential health issues. Diabetes is a particularly relevant condition in this regard, given the fact that it is a chronic disorder with lifelong impact [13,14]. Individual decision-making is driven by context which is very distinct in the health domain. This was clearly demonstrated in our previous work analyzing the role of social and family context and personal choice profiles [14]. Compliance with prescribed treatments can be interpreted as health investment, which is very different from contextual decision in the economic domain. In both cases, past experiences updated by feedback and emotional processes play an important role. This is in line with the suggestion of Tarrant et al. [15], who claimed that the study of patients’ decisions to comply and collaborate in doctor-patient interactions can be envisaged within the framework of interactive decision-making and economic utility models. This proposal inspired our study.

Studies of risk-taking and feedback processing in social interactions can be extended beyond the economic domain. It is relevant to consider these concepts also when people engage in risky health behavior and lack avoidance of future complications with high probability. In general, this type of research in traditional approaches highlights individual differences in proneness to maladaptive behavior [3] or suboptimal economic decisions during a repeated interaction trust game in which participants learn to expect different monetary returns through trial-to-trial feedback to choose the most advantageous way to invest. In other words, participant investment (option selection) is based on positive or negative feedback, because the participant expects fair treatment. Similarly, social collaboration or prosocial behavior is less likely to occur continuously if other’s behaviors are perceived as unfair or result from norm violation [16,17].

Importantly, successful decision-making under uncertainty requires adaptive learning, requiring as the “ability to estimate expected uncertainty” [18]. The variability of outcomes affects the capability to correctly infer probabilistic models. The learning rate is based on the computation of difference between the expected value and the real outcome, called the reward prediction error (RPE). Methodologically, using different reward magnitudes associated with different probability distributions (same mean reward) and with a fixed relative uncertainty from trial to trial allows estimation of the expected uncertainty (standard deviation of a reward distribution–taken as risk) [19]. Furthermore, as participants do not know outcome probabilities in advance, their initial decisions are made under complete ambiguity, meaning that that they can learn through feedback [20].

We aimed at extending economic utility-based models to decision-making within the health domain. Participants (91 adults with Type 1 Diabetes) completed two experimental interactive neuroeconomic game tasks, namely, trust games with decision-making under uncertainty in matched economic and health contexts. As decision-making is suggested to be strongly context dependent [21] we asked the question whether different decision-making profiles emerge from economic and health tasks.

We expected that decision-making profiles would be associated with the quality of metabolic control in diabetes. Better ability to learn health attitudes, leads to better compliance and therefore to better metabolic control. We hypothesized that compliant (trustworthy in dyadic interactions) patients have better metabolic control than non-compliant patients. Furthermore, we hypothesized that both groups (with adequate and non-adequate metabolic control) can learn context contingencies in all tasks, but the control group (with good metabolic control) will consider updated values when they are selecting an option, while no significant switching is expected in the group without metabolic control. Third, we aimed to investigate how patient collaboration (health choice) changes with different feedback (patterns of doctor-patient interaction) in a trial-and-error feedback processing paradigm.

## 2. Materials and Methods

Written informed consent forms were signed by all subjects after an explanation of the nature and duration of the study. It was approved by the Ethics Committee of the Faculty of Medicine of the University of Coimbra, in accordance with the Declaration of Helsinki.

### 2.1. Sample Characterization

A total of 91 volunteers from University Hospital, referred to the clinical assessment of the Department of Endocrinology, Diabetes and Metabolism at the University Hospital of Coimbra, Portugal (EDM), were divided in 2 groups according to the dynamics of HbA1c values over time: 42 patients with no glycemic control (mean age: 36.19 ± 8.67, mean educational level: 1.36 ± 0.075) and 49 patients (clinical control group) with glycemic control (mean age: 37.20 ± 9.47; mean educational level:1.65 ± 0.07). We focused on the comparison between clinical groups with or without metabolic control. By definition, healthy participants have unchanged and preserved metabolic control. Because metabolic status is considered stable in patients with glycemic control (clinical control group), performance results from a healthy control group (N = 53) are normative and presented as supplementary material (Tables S1–S4).

Table 1 summarizes the groups’ demographic, cognitive/neuropsychological, clinical characteristics, and risk measures. It shows the most relevant data (for more details see below). Groups are matched for age, gender, and civil status. All these patients had a similar access to and level of medical care with periodic 6 months consultations at the same hospital and similar medication protocols. All these patients had medical devices to monitor glucose levels and medication was being adjusted accordingly. Clinicians involved in the consultation at the University Hospital evaluated current and past symptoms and complications. Body Mass Index (BMI) and biochemical data were also collected. To divide patients into groups with or without successful metabolic control, values of HbA1c for the patient consultation history over multiple time points were collected. For the first group (Metabolic Control-MC), we included (1) patients with continuously descending and improving values of HbA1c over time, (2) patients with low (normal) stable/invariant values that did not change beyond 0.5 and (3) patients whose values varied more than 0.5, but the maximum value of this oscillation was lower than 8.0 (64 mmol/mol). For the second group (No Metabolic Control-NoMC), we included patients with the following dynamic profiles: (1) continuously ascending values of HbA1c over the time, (2) patients with high (abnormal) stable values that did not change beyond 0.5 over the time and (3) patients whose values varied more than 0.5, but the minimum value of this oscillation was more than 8.0.

Participants were asked to completed a number of self-reported questionnaires, validated to the Portuguese population, to characterize the sample: the Eysenck Personality Questionnaire (EPQ) (Portuguese version from [22]) to evaluate personality traits; Behavior Impulsivity Scale-11 (BIS-11; translated by [23]; validation for the Portuguese population by [24]) to evaluate impulsivity in general; DOSPERT ([21,25]; Portuguese translation by [26]) for individual perception of risk taking assessment in economic and health domains; past and present risk taking to evaluate variations of risk profile across the life span; an intertemporal choice questionnaire, where participants were asked to choose one of three options that differ in risk or sacrifice to delay reward for three different contexts, economic, general health and diabetes specifically [27] (see Supplementary material). In the economic context, participants had to choose between three levels of wait time to win money: more time, more money. In general health, participants had to choose between three drugs to avoid possible heart infarct: more effective, more secondary effects. In diabetes context, participants had to choose between three levels of number of insulin therapeutic pricks (the larger the number, the better the investment in metabolic control–more pricks corresponding to delay in long term ocular complications (see Supplementary information). Finally, the Dutch Eating Behavior Questionnaire (DEBQ) [28,29] was administered to evaluate three types of eating styles: restrained (avoid eating more than was initially defined), external (to eat motivated by external factors such as good food smell and how it looks) and emotional (to eat in response to emotions). Participants also performed cognitive tests with Portuguese population norms, to verify if they could be included in this study: fluid intelligence (Raven Progressive Matrices) [30], crystalized intelligence (Vocabulary of WAIS-III) and executive functions such as attentional processes and working memory (Digits Forward and Backward subtests of WAIS-III) [31]. Participants aged more than 50 filled out MOCA (Montreal Cognitive Assessment) [32].

Exclusion criteria were: other people in the nuclear family diagnosed with diabetes for at least one year and other current major chronic disease, evidence for past or current history of neurological and psychiatric disorders, recent diseases, major medical illness (cancer, anaemia, and thyroid dysfunction) and severe visual or hearing loss. In total, 2 patients were excluded, having presented a history of psychiatric disorder.

### 2.2. Experimental Interactive Game Decision-Making Tasks

**Figure 1:**
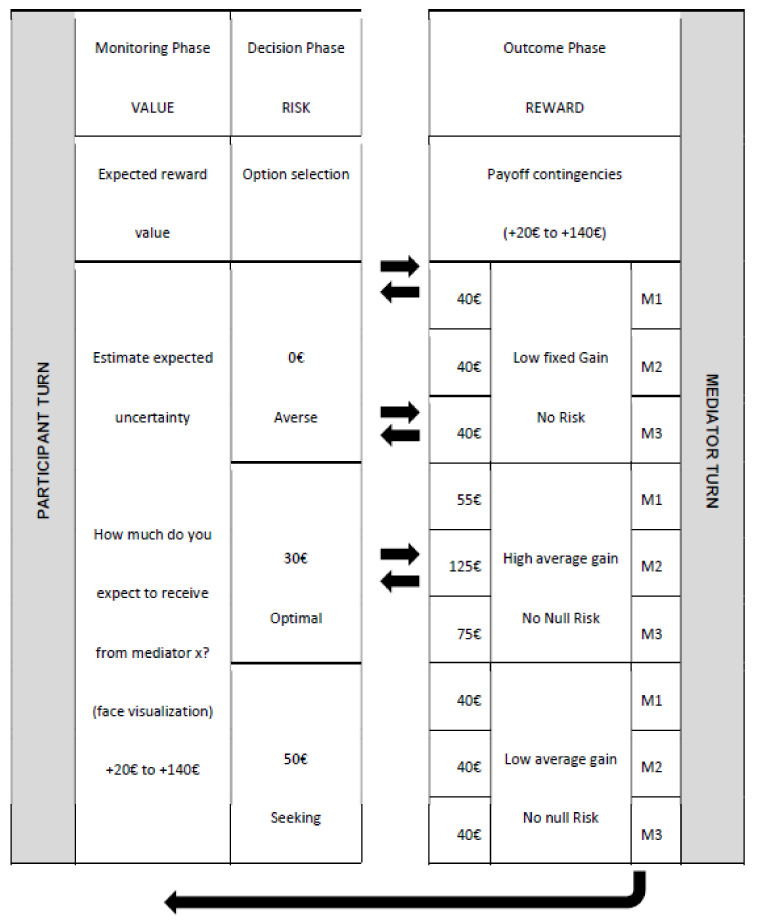
Example of economic experimental design considering a run sequence in trustor-trustee interaction. M0—the non-human mediator—has the same reward contingencies as M1. For details see text.

**Figure 2:**
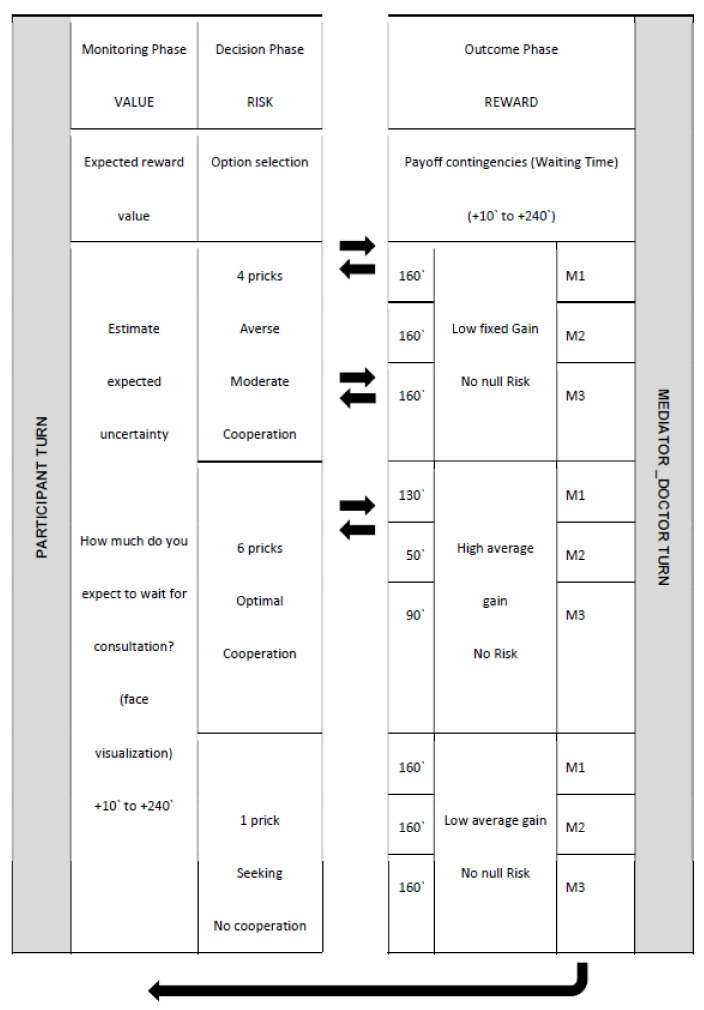
Example of health experimental design considering a run sequence in doctor-patient interaction. For details see text.

As in game theory, each player has a way of acting; the strategy, and actions of two or more decision-makers lead to option selection [33]. To mimic this situation, we presented two experimental interactive games, named: 1. Computer and Human Mediator Neuroeconomics Experiment and 2. Health Context Interaction Experiment (inspired by the neuroeconomics framework) (see Figure 1 and Figure 2).

Risky behavior in the health context is an option among others with uncertain probabilistic consequences (leading to heath preservation or loss) while in the economic domain risky behavior is understood as a statistical uncertainty expressed as variance in monetary gain or losses [34]. The first experiment refers to situations without a medical context and the second is a tailored task with a medical risk and reward value. It was played in iterated form, where the game is made up of several rounds (runs), repeated 7 times between each type of players. At each trial, participants know with whom they are playing through face recognition of the mediator in that run. All trials, 21 in total, require that participants press one of three buttons to indicate their action selection.

Experiment 1: Computer and Human Mediator Neuroeconomics Experiment (Economic Trust Game)

The first game is a classic neuroeconomic experiment and it helps define risk profiles. Participants’ challenge during this trust game was to learn the optimal investment choice based on three mediator’s outcomes. Within three distinct risk alternatives (0 €, 30 € or 50 €), they had to choose one (option selection), to wait for the respective outcome (feedback) and to indicate how much money they expect to receive from that mediator in the next run (estimate expected uncertainty). Mediator 1 has a low range for reward. Mediator 2 has an extreme range, reinforcing optimal decision. Mediator 3 has a moderate range, mid-way between the M1 and M2 profile (trust investment is reciprocated in a moderate way). Outcome reward was also different according to the participant’s option (0, 30 or 50 euros) for all mediators: 1. For the “0” option (no risk investment), the participant received a known low fixed gain (40 euros). 2. For the “50 euros” option (risk investment), a low average gain was offered (same mean reward, (40 euros) that could vary from 20 to 60 euros. 3. For the “30 euros” option (adjusted risk) the average gain that was earned depended on the mediator, who gave a low, extreme or moderate reward: Mediator 1 “35–75 euros“ Mediator 2 “100–140 euros”; Mediator 3 “55–95 euros”. All of them have the same interval (range of 40). We also introduced a computer mediator (MO) with the same reward contingencies as M1. Participants were exposed in alternation during the 7 runs for each mediator. Eachoutcome pattern differed in terms of reward distribution (low, moderate, or extreme) for optimal choice. More specifically, each trial was divided into three phases: monitoring phase, decision phase and outcome phase (Figure 1).

The experiment began with the monitoring phase: the subjects had to indicate an expected value (euros gain or loss) for the next trial which varied between +20 to +140, answering the template question. At the first trial, as the participant did not know each mediator’s payoff contingencies, we could obtain the initial risk profile and learn how the subject initially performed with each mediator (presence or absence of game strategy/planning). Participants had to remember past feedbacks (outcomes) to update the expected value and decide the next investment for each mediator (estimated expected uncertainty). In that way, we could gather empirical evidence to support different psychological profiles of rational decision-making.

Experiment 2: Extending Utility Based Neuroeconomics to the Health Context (Health Trust Game)

The health context interaction experiment, inspired by the classical neuroeconomics experiment, used clinical human mediators. In this second game, we adapted previous experiments to the health context and added a rule/norm: more patient cooperation allowed less waiting time to consultation (less waiting time meaning larger reward). So, we presented one of three different clinicians one at a time, in order to represent three different types of human mediator feedback as in Game 1 (Low, Moderate and Extreme Rule Following) for optimal choice. Mediator 1 has a low range for reward. Mediator 2 has an extreme range, reinforcing optimal decision fulfilling the pre-established rule. For this mediator, the average waiting times is lowest, precisely because of the reinforcement levels. Mediator 3 has a moderate range, between the M1 and M2 profiles. Outcome rewards also differed according to participant option (1, 4 or 6 pricks) for all mediators: 1. For “4” option (moderate cooperation), a known low fixed gain (160′) was received. 2. For “1” option (no cooperation), a low average gain was offered (same mean reward, 160′ but it could vary from 10 to 240 min). 3. For “6” option (highest cooperation), a high average gain was earned, depending on the mediator: Mediator 1 “90–170” Mediator 2 “10–90”; Mediator 3 “50–130”. All of them have the same interval “80”.

In the first phase we presented different health impact levels of developing negative symptoms (for example, diabetic foot) due to impaired glycaemic control. Subjects choose to cooperate (health investment) or not by accepting several therapeutic needle pricks (1 prick meaning no cooperation; 4 pricks meaning medium cooperation; 6 pricks meaning highest cooperation) without prior knowledge of the priority reward (amount of time needed to wait for consultation). So, 6 pricks means that the participant is accepting a more intensive insulin therapy (more injections), which means more investment in the health domain. The final priority outcome rank is in parallel with the final monetary outcome Neuroeconomic Game 1. Note that in this case priority for being received corresponds to low amount’s (less time) and in turn to a better outcome. Priority is defined by the number of minutes needed to wait before a consultation (0 to 260). In this game, a computer mediator (available from experiment 1) was not used (Figure 2).

### 2.3. Data Analysis

Data were analyzed using IBM SPSS Statistics (v24) [35]. Descriptive statistics are reported as mean ± SD. Prior to analysis, raw data were examined for normality by the Shapiro–Wilks goodness-of-fit test [36]. Null-hypothesis statistical tests were evaluated according to an alpha value of *p* = 0.05. The chi-square test was used to compare categorical variables, and nonparametric tests (Kruskal-–Wallis) were used to compare ordinal variables. To assess possible between group-differences from expected value, investment and feedback, data were submitted to independent sample t-tests. Non-parametric tests, such as Friedman tests, were applied to analyze differences between expected value, investment, and feedback within each group for the economic and health context, searching for post-hoc differences between mediators. We performed Friedman tests to investigate the main effects of the experimental 7 runs for each mediator (M0, M1, M2 and M3) and subsequent post-hoc comparisons. Paired t-tests were performed to investigate whether participants learned the optimal choices, comparing feedback and expected values means for each mediator in different groups.

## 3. Results

We investigated the role of context (economic and health) and risk behavior patterns in diabetic patients as a function of group profile (with and without metabolic control) considering initial decision options and their subsequent update through sequential learning.

Interestingly, self-report measures showed important behavioural differences of the groups defined by the biological division of metabolic control (for details see Table 1). The group lacking metabolic control showed higher levels of impulsivity, lack of planning, low perception of health risk, high past and present risk, and intermediate (lower) rewards for health intertemporal choice (more secondary effects). Scales of emotional and external eating behaviour were also significantly different between groups with more external eating behavior for the NoMC group.

Concerning the experimental tasks assessing choice behavior under uncertainty and initial game strategy, we examined the initial risk profile as assessed by initial Decision Phase results. Thereafter, we investigated how participants adjusted decision-making (choice impact) if probabilistic learning feedback was accomplished. For learning achievement, we measured differences on the Expected Value for each mediator, according to mediator feedback payoff contingencies. In this way, we were able to verify if there were different risk profiles according to context and groups and make inferences about learning probabilities and their impact on investment, particularly in the health context, which featured different patterns of doctor-patient interactions in patient compliance. Compliance in this context is seen as a personal investment in health. Table 2 presents results from descriptive statistics of expected value, investment and feedback depending on each mediator (M0, M1, M2 and M3) for both groups in the economic and health context. The mean difference between feedback and expected values for each mediator in both groups is not statistically significant (suggesting good predictive ability) except in the economic context for M2 (NoMC and MC group) and M3 (NoMC group): M2-NoMC mean difference = 33.85; SD = 25.27; t (41) = 5.97, *p* < 0.05; M2-MC group mean difference = 31.90; SD = 27.91; t (48) = 6.14 *p* < 0.05; M3-NoMC group mean difference = 39.72; SD = 24.61; t (41) = 11.15, *p* < 0.05.

### 3.1. Decision-Making under Uncertainty (The First Play Move)

Considering the first play move, we observed distinct profiles. The group with preserved metabolic control (MC) showed a consistent behavior across both contextual tasks and initial strategy (similar investment for all mediators at the first play move, with planning investment). There is an association between initial strategy for both contexts in the MC group [x^2^ (1) = 5.38, *p* = 0.02]: subjects tended to be strategically consistent (if they invested the same with all mediators in the economic task, they used the same procedure in the clinical task). We did not find an association between initial strategy for both tasks in the NoMC group (no planning investment).

### 3.2. Adjusted Decision-Making during Probabilistic Learning (Sequential Play Move)

Friedman tests showed a significant main effect of mediator concerning Expected Values, Investment and Feedback, for both groups in both tasks. The exception was that in the MC group there was no mediator effect for investment in the health task. Posthoc tests showed that sensitivity to mediators stemmed mainly from mediators M2 and M3 (the ones that show clear feedback differences in the trust games). (For details see Supplementary material, Table S4).

Concerning changes during the tasks, subjects were able to learn each mediator’s profile (Monitoring Phase) presenting differences in expected values according to feedback on mediator contingencies, expecting to receive more money (economic task) and less waiting time (health related task) from Mediators 2 and 3 (Table 1).

Despite being able to learn mediator feedback contingencies, groups differed in their options for investment in economic and health domains. According to Table 3, patients without metabolic control chose to invest in mediator 3 (M3) whereas in the health context they opted finally for collaboration with Mediator 2 (the clinician who did not violate the norm and follow the rule: high compliance less waiting time). In turn, patients with adequate metabolic control (Table 4), revealed no significant preference (or only very marginal) for investment in both contexts. Interestingly, in the health context they opted to collaborate regardless of the doctor payoff contingencies.

## 4. Discussion

The main aim of this study was to investigate the role of health context (defining patterns of risky behavior) in decision-making under uncertainty in clinical groups where such decisions are extremely relevant, such as in diabetes. This was achieved using trust games, going beyond traditional economic utility-based tasks to health context. By separating different stages of the decision-making process, we gained evidence about feedback processing (update) and how groups differ in considering these update values on subsequent investment. Finally, our findings provide insight into a special form of social decision-making based on patient-doctor interactions and how different payoff contingencies influence differently the collaboration by patients with and without glycemic control. This enabled us to directly relate decision-making profiles to biological status.

Our results extend prior evidence that human decision-making is context dependent [21] in the health domain, while providing clues for its relation with biological outcomes. Different decision-making profiles emerged from both economic and health tasks. In the same way, different decision-making profiles emerged from categorical differences in the quality of metabolic control.

In initial strategy for investment, each group behaved differently, showing that strategy and planning were related to the adequacy level of metabolic control. Considering iterative decision making, groups also behaved differently according to context even though they dealt with the same disease (allowing for group matching while differentiating biological outcome). In general, both groups were able to detect payoff contingencies (incorporate feedback processing, updating the experience [37]). For M2 in economic contexts it seems that the large amount of reward is still a positive surprise and this may why participants expected less than they received on average, in this particular case. However, regarding the health domain, patients without metabolic control seem to be more dependent on external reinforcement than the glycemic control group. Our results suggest that they tend to be more sensitive to putative social norm violations in the clinical setting because patient collaboration changed when faced with different doctors’ payoff contingencies. These results reveal that patients without metabolic control are not indifferent to the patient-clinician relationship, which is a somewhat reassuring finding from this study, despite the non-compliant profile. In contrast, MC patients seem to keep taking good health decisions, right from the start, independently of payoff contingencies. Therefore, compliant patients had good metabolic control as we expected. Our pattern of studies could be linked to the statement of Gray et al. [38]: “A patient may become ‘stuck’ with a doctor in whom he or she lacks confidence”, with a direct impact on adherence. For clinical practice, this requires counteracting health providers’ desire to withdraw when a patient persists in maladaptive behavior, preventing a cycle of non-cooperation [39].

Our data supports the notion that risk taking behavior profiles can lead to distinct levels of outcome for a biological variable (in our case glycemic control), suggesting distinct mechanisms of behavioral control. This implies that early detection of these behavioral profiles can enable swift intervention approaches to improve compliance and prevent complications.

## 5. Limitations

The role of the type of human mediator in the experimental games could not be fully explored because this would require a larger sample size. With our experiment, differences in decision phase (investment) were due to context and a biological variable (HbA1C, a measure of metabolic control), but it remains unclear if there are other mediator variables or if contextual cues are ignored or salient depending on other variables.

Further evidence for matching of economic and health related tasks would also be valuable, to show that these tasks share similar neural correlates. Neuroimaging studies could be advantageous to further demonstrate the ecological validity of this approach.

## 6. Future Directions

Despite these shortcomings, the current study could guide future studies on dyadic interactions (family members and patient adherence) and neuroimaging approaches. These studies will be helpful to understand the neural correlates of prediction error (High/Low Expected Value versus Low/High Feedback); the neural correlates on how updated values could explain shifting decisions (High/Low Feedback versus High/Low Investment) and to investigate the neural basis of risk perception and risk taking (High/Low Expected Value versus Low/High Investment). Finally, further studies could validate intervention programs that promote treatment adherence with training of socioaffective and interaction skills [40].

## 7. Conclusions

Through modelling interactive trust games and translating them into the health domain, our findings suggest a strong role of context and biological status in decision-making under uncertainty since different decision-making profiles emerged between patients with and without metabolic control. Furthermore, by distinguishing different stages of the decision-making process (monitoring, decision, and outcome) we were able to disentangle feedback processing from choice itself, getting evidence that probabilistic learning is not enough to explain decision-making in these contexts and groups. Our findings also contribute to better understanding howpatient collaboration varies in function of perceived social norm violation in the health domain, highlighting a biologically determined decision-making profile and, consequently, providing information that could guide adherence to treatment programs with clinical implications.

## Figures and Tables

**Table 1:** Demographic characteristics, cognitive results, and relevant clinical features and self-report risk measures for NoMC and MC groups (N = 91).

Variables	MC (N = 49)	NoMC (N = 42)	X^2^	t	U	gl	*p*	d
Demographic data								
Gender (M/F)	31/18	25/17	0.134	-----	-----	-----	0.824	0.07
Age (y)	37.20 (9.47)	36.19 (8.67)	-----	0.529	-----	89	0.59	−0.11
Civil State (Single/Couple)	22/27	24/18	1.367	-----	-----	1	0.244	0.07
Household members (1/2/3)	17/28/3	16/21/5	1.695	-----	-----	1	0.428	0.08
Household income B (1/2)	33/15	16/26	8.94	-----	-----	1	0.003	0.66
Residence	20/12/16	16/17/9	2.97	-----	-----	2	0.226	0.36
Education level (1/2)	17/32	27/15	7.93	-----	-----	1	0.005	0.61
Cognitive data								
Vocabulary	32.33 (3.47)	33.60 (2.81)	-----	-----	807	-----	0.075	0.034
Digit Memory	14.82 (2.15)	14.10 (1.92)	-----	-----	1273	-----	0.05	0.416
RPMT	8.04 (0.90)	8.05 (1.01)	-----	-----	981	-----	0.688	0.08
Clinical features								
Disease onset (</>18)	24/25	24/18	0.605	-----	-----	1	0.382	0.16
Disease Dealing Time	17.56 (10.38)	17.21 (9.58)	-----	−0.161	-----	89	0.870	−0.034
HbA1c(%/mmol/mol)	7.19/55 (0.65)	8.52/70 (1.22)	-----	6.329	-----	89	<0.001	0.07
BMI	24.95 (3.31)	25.20 (3.81)	-----	-----	989	-----	0.750	0.067
Complications (Y/N)	21/28	30/12	7.94	-----	-----	1	0.006	0.62
Smoking status (Y/N)	11/38	7/35	0.48	-----	-----	1	0.49	0.14
Self-report measures								
Neuroticism	6.49 (4.02)	9.95 (4.22)	-----	4.005	-----	89	<0.001	0.84
Extroversion	13.12 (3.49)	10.98 (3.61)	-----	−2.88	-----	89	0.005	−0.61
Impulsivity	54.11 (7.06)	58.05 (8.03)	-----	2.138	-----	89	0.035	0.45
Lack of planning	14.32 (3.76)	17.03 (4.41)	-----	-----	657.5	-----	0.003	3.34
Health risk perception	37.65 (5.25)	35.98 (8.8)	-----	-----	1273	-----	0.029	0.41
Past Risk	14.60 (3.73)	12.00 (3.29)	-----	3.52	-----	89	0.001	0.74
Present Risk	10.67 (2.80)	13.64 (4.31)	-----	3.83	-----	89	<0.001	0.81
Health Intertemporal Choice	25/15/9	13/24/5	6.51	-----	-----	2	0.039	0.55
Emotional Eating Behavior	2.34 (0.54)	2.29 (0.78)	------	2.84	-----	89	0.006	0.59
External Eating Behavior	2.34 (0.54)	2.58 (0.51)	------	2.10	-----	89	0.039	0.44

Educational level (1 = 12 years, secondary education; 2 = university degree or higher; Household income (1 = stable; 2 = unstable); Members of the household (1 = living alone; 2 = living as a couple; 3 = living with children); Residence as distance to health services, in travel time (1 = Coimbra; 2 = <1 h; 3 = >1 h) RPMT = Raven’s Progressive Matrices Tests; BMI = body mass index. Health Intemporal choice (longer and larger reward; intermediate reward; small sooner reward).

**Table 2:** Descriptive statistics, using the experimental outcome variables for either economic or health contexts in both groups (No-MC, no metabolic control achieved; MC–successful metabolic control). These are sorted in terms of expected value, investment and feedback values.

	Economic Context			
	NoMC		MC	
Variable	M	SD	M	SD
Expected Value				
M0	66.96	21.55	64.13	19.47
M1	63.64	20.91	67.88	18.41
M2	72.91	24.42	74.90	22.64
M3	67.08	25.58	61.96	27.19
Investment				
M0	37.41	23.59	36.56	18.73
M1	36.83	19.42	39.64	17.71
M2	40.82	21.16	40.07	16.44
M3	55.97	31.26	54.88	33.28
Feedback				
M0	75.55	18.53	73.95	16.52
M1	73.28	18.36	79.56	18.36
M2	106.86	38.14	106.80	33.94
M3	106.80	33.94	71.83	29.43
	**Health Context**			
	NoMC		MC	
Variable	M	SD	M	SD
Expected Value				
M1	125.53	23.55	116.81	28.22
M2	106.70	26.26	96.70	36.67
M3	106.99	27.16	105.62	29.32
Investment				
M1	4.68	0.76	4.82	0.86
M2	5.17	0.72	5.10	0.86
M3	4.87	0.85	4.87	1.04
Feedback				
M1	149.14	17.52	144.95	14.22
M2	98.13	37.69	97.42	36.21
M3	125.91	22.51	119.08	26.59

**Table 3:** A Repeated measures comparison of investment during the 7 runs for each type of human mediator (M1–M3), to investigate learning of mediator feedback contingencies (Friedman non-parametric test) on economic and health related context experimental tasks for patients without metabolic control. ** *p* value < 0.05.

NoMC Group								
Variable	Economic Context (N = 42)				Health Related Context (N = 42)			
	Friedman	gl	*p*	W	Friedman	gl	*p*	W
Investment								
M0 (1–7)	7.23	6	0.300	0.03				
M1 (1–7)	4.86	6	0.560	0.02	7.29	6	0.294	0.03
M2 (1–7)	7.14	6	0.308	0.03	17.85	6	0.007 **	0.07
M3 (1–7)	14.13	6	0.028 **	0.60	7.79	6	0.254	0.03

**Table 4:** Repeated measures comparison of investment during the 7 runs for each type of human mediator (M1–M3), to investigate learning of mediator feedback contingencies (Friedman non-parametric test) on economic and health related context experimental tasks for patients with metabolic control.

MC Group								
Variable	Economic Context (N = 49)				Health Related Context (N = 49)			
	Friedman	df	*p*	W	Friedman	df	*p*	W
Investment								
M0 (1–7)	12.76	6	0.050	0.05				
M1 (1–7)	10.54	6	0.104	0.10	2.53	6	0.865	0.03
M2 (1–7)	6.86	6	0.334	0.02	5.57	6	0.473	0.02
M3 (1–7)	12.47	6	0.052	0.04	2.53	6	0.860	0.01

## Data Availability

Data can be provided by the corresponding author upon reasonable request. Please contact cibit@uc.pt.

## Supplementary Materials

The following supporting information can be downloaded at: https://www.mdpi.com/article/10.3390/jpm12081236/s1, Table S1. Demographic Characteristics, Cognitive results, personality, self-report risk measures and eating behavior for healthy participants; Table S2. Descriptive statistics on economic and health context experimental task for healthy participants; Table S3. Repeated measure comparison (Friedman Non-parametric test) on economic and health related context experimental tasks for healthy participants; Table S4. (A) Repeated measures comparison between each mediator for Expected Value, Investment and Feedback (Friedman Non-parametric test for main effects of mediator and posthoc tests) on economic and health related context experimental tasks for NoMC and MC groups; (B) Posthoc tests for each variable (expected, investment and feedback) on economic and health related context experimental tasks for NoMC and MC groups.

## References

[1] Charness G., Gneezy U., Kuhn M.A. (2012). Experimental Methods: Between-Subject and Within-Subject Design. J. Econ. Behav. Organ..

[2] Glimcher P., Porris M., Gazzaniga M., Norton W.W. (2004). Neuronal Studies of Decision-Making in the Visual-Saccadic System. The Cognitive Neuroscience.

[3] Lane S., Cherek D.R. (2000). Analysis of Risk Taking in Adults with A History of High-Risk Behavior. Drug Alcohol Depend..

[4] Mohr P., Biele G., Heekeren H. (2010). Neural Processing of Risk. J. Neurosci..

[5] Ruff C., Huettel S., Glimcher P.W., Fehr E. (2014). Experimental Methods in Cognitive Neuroscience. Neuroeconomics: Decision-Making and the Brain.

[6] Christopoulos G.I., Tobler P.N., Bossaerts P., Dolan R.J., Schultz W. (2009). Neural Correlates of Value, Risk and Risk Aversion Contributing to Decision-Making Under Risk. J. Neurosci..

[7] Kim S.H., Yoon H., Hamann S. (2015). Individual Differences in Sensitivity to Reward and Punishment and Neural Activity during Reward and Avoidance Learning. Soc. Cogn. Affect. Neurosci..

[8] Rangel A., Camerer C., Montague R. (2008). A Framework for Studying the Neurobiology of Valed-Based Decision Making. Nat. Rev..

[9] Bechara A., Damasio H., Tranel D., Damasio A.R. (1997). Deciding Advantageously Before Knowing the Advantageous Strategy. Science.

[10] Dong G., Zhang Y., Xu J., Lin X., Du X. (2015). How the Risky Features of Previous Selection Affect Subsequent Decision-Making: Evidence from Behavioral and Fmri Measures. Front. Neurosci..

[11] Megías A., Cándido A., Maldonado A., Catena A. (2018). Neural Correlates of Risk Perception as a Function of Risk Level: An Approach to the Study of Risk through a Daily Life Task. Neuropsychologica.

[12] Vives M.-L., FeldmanHall O. (2018). Tolerance to Ambiguous Uncertainty Predicts Prosocial Behavior. Nat. Commun..

[13] Rustad J.K., Musselman D.L., Skyler J.S., Matheson D., Delamater A., Kenyon N.S., Cáceda R., Nemeroff C.B. (2013). Decision-Making in Diabetes Mellitus Type 1. J. Neuropsychiatry Clin. Neurosci..

[14] Jorge H., Duarte I.C., Correia B.R., Barros L., Relvas A.P., Castelo-Branco M. (2021). Successful Metabolic Control in Diabetes Type 1 Depends on Individual Neuroeconomic and Health Risk-Taking Decision Endophenotypes: A New Target in Personalized Care. Psychol. Med..

[15] Tarrant C., Stokes T., Colman A.M. (2004). Models of the Medical Consultation: Opportunities and Limitations of A Game Theory Perspective. BMJ Qual. Saf..

[16] Delgado M.R., Frank R.H., A Phelps E. (2005). Perceptions of Moral Character Modulate the Neural Systems of Reward during the Trust Game. Nat. Neurosci..

[17] Zinchenko O., Arsalidou M. (2017). Brain Responses to Social Norms: Meta-Analyses of f MRI Studies. Hum. Brain Mapp..

[18] Soltani A., Izquierdo A. (2019). Adaptive Learning under Expected and Unexpected Uncertainty. Nat. Rev. Neurosci..

[19] Li Y., Dudman J.T. (2013). Mice Infer Probabilistic Models for Timing. Proc. Natl. Acad. Sci. USA.

[20] Platt M.L., A Huettel S. (2008). Risky Business: The Neuroeconomics of Decision Making under Uncertainty. Nat. Neurosci..

[21] Blais A.R., Weber E.U. (2006). A Domain-Specific Risk-Taking (Dospert) Scale for Adult Populations. Judgm. Decis.-Mak..

[22] Castro-Fonseca A., Eysenck S.B., Simões A. (1991). Um Estudo Intercultural da Personalidade: Comparação de Adultos Portugueses e Ingleses no EPQ. Rev. Port. Pedagog..

[23] Cruz A., Barbosa F. (2012). European Portuguese Version of BIS-11 for Research. http://www.impulsivity.org/measurement/BIS11_Portuguese.

[24] Fernandes D. (2014). Validation Studies of Barratt Impulsivity Scale BIS-11-for Portuguese Population. Master’s Thesis.

[25] Weber E.U., Blais A.R., Betz N.E. (2002). A Domain-Specific Risk-Attitude Scale: Measuring Risk Perceptions and Risk Behaviors. J. Behav. Decis. Mak..

[26] Silva J.P. (2012). Risk Profiling and the DOSPERT Scale: An Approach Using Prospect Theory. Master’s Thesis.

[27] Fernie G., Cole J.C., Goudie A.J., Field M. (2010). Risk-Taking but not Response Inhibition or Delay Discounting Predict Alcohol Consumption in Social Drinkers. Drug Alcohol Depend..

[28] Van Strien T., Frijters J.E.R., Bergers G.P.A., Defares P.B. (1986). The Dutch Eating Behavior Questionnaire (DEBQ) for Assessment of Restrained, Emotional and External Eating Behavior. Int. J. Eat. Disord..

[29] Viana V., Sinde S. (2003). Eating style: Adaptation and validation of the Dutch eating behavior questionnaire. Psicol. Teor. Investig. Prat..

[30] Simões M., Almeida L.S., Simões M.R., Machado C., Gonçalves M.M. (2004). Critical Recension: Raven’s Coloured Progress. Matrices test in Potugal. Avaliação Psicológica: Instrumentos Validados para a População Portuguesa [Psychological Assessment: Instruments Validated for Portuguese Population].

[31] Wechsler D. (2008). WAIS-III- Wechsler Adult Intelligence Scale.

[32] Freitas S., Simoes M., Alves L., Santana I. (2011). Montreal Cognitive Assessment (MoCA): Normative Study for the Portuguese Population. J. Clin. Exp. Neuropsychol..

[33] Moallen N.R., Ray L.A. (2012). Dimensions of Impulsivity among Heavy Drinkers, Smokers, and Heavy Drinking Smokers: Singular and Combined Effects. Addict. Behav..

[34] Schultz W., O’Neill M., Tobler P., Kobayashi S. (2011). Neuronal Signals for Reward Risk in Frontal Cortex. Ann. N. Y. Acad. Sci..

[35] Maroco J. (2007). Statistical Analysis: Using SPSS.

[36] Ghasemi A., Zahediasl S. (2012). Normality Tests for Statistical Analysis: A Guide for Non-Statisticians. Int. J. Endocrinol. Metab..

[37] O’Doherty J., Cockburn J., Pauli W. (2017). Learning, Reward and Decision-Making. Annu. Rev. Psychol..

[38] Gray D.P., Evans P., Sweeney K., Lings P. (2003). Towards a Theory of Continuity of Care. J. R. Soc. Med..

[39] Rilling J.K., Gutman D.A., Zeh T.R., Pagnoni G., Berns G.S., Kilts C.D. (2002). A Neural Basis for Social Cooperation. Neuron.

[40] Singer T., Tusche A., Glimcher P.W., Fehr E. (2014). Understanding Others: Brains Mechanisms of Theory of Mind and Empathy. Neuroeconomics. Decision-Making and the Brain.

