# Supplementary material for: Trust-Based Decision-Making in the Health Context Discriminates Biological Risk Profiles in Type 1 Diabetes
Source: J Pers Med. 2022 Jul 28;12(8):1236. doi: 10.3390/jpm12081236 (PMC9410470; doi:10.3390/jpm12081236)
Supplement: Supplementary file 1 [file jpm-12-01236-s001.zip › jpm-1790626-supplementary.pdf]

Supplementary Information

Intertemporal choice questionnaire

Delay discounting in three domains: economic, general health and diabetes context. It means a preference for smaller rewards versus larger delayed rewards because the subject value of an outcome decrease as the time to its receipt increases.

Economic context:

Image you receive an inherence of 1000 euros. You have three options. What do you prefer?

1.You can decide to receive it in 2 years twice (2000 euros).

2 You can decide to receive it in 5 years, 5x 1000 (5000 euros).

3.You can decide to receive it in 9 years, 9x1000 (9000 euros)

General Health:

Image you had a severe chest pain. At hospital, clinicians told you that you are at risk of stroke. You have three options of therapeutic drugs. What do you prefer?

1.You can decide to take drug A, it causes nausea and vomiting but prevents angina pectoris in 9 years.

2 You can decide to take drug B, it causes vomiting but prevents angina pectoris in 5 years.

3.You can decide to take drug C, it causes nausea but only prevents angina pectoris in 2 years.

Diabetes:

Image that after an eye exam, clinicians told you that you are in danger of blinding. You have three options. What do you prefer?

1.You can decide to take 5 daily pricks and prevent visual impairments in 15 years.

2 You can decide to take 2 daily pricks and prevent visual impairments in 10 years.

3.You can decide to take 0 daily pricks and prevent visual impairments in 5 years.

Table S1.

Demographic Characteristics, Cognitive results, personality, self-report risk measures and eating behavior for healthy participants

Variables	Health Control Group (N=53)
Demographic data	
Gender (M/F)	27/26
Age (y)	35.66(8.51)
Civil State (Single/Couple)	22/31
Household members (1/2/3)	16/32/5
Household income B (1/2)	25/28
Residence	53/0/0
Education level (1/2)	4/49
Cognitive data	
Vocabulary	32.19 (3.15)
Digit Memory	16.57(2.83)
RPMT	8.21(0.82)
Self-report measures	
Neuroticism	7.55(3.99)
Extroversion	11.87(3.58)
Impulsivity	57.64(7.64)
Lack of planning	15.62(4.01)
Health risk perception	35.91(7.59)
Past Risk	14.33(3.80)

Present Risk	12.38(3.71)
Health Intertemporal Choice	23/13/25
Emotional Eating Behavior	1.96(0.91)
External Eating Behavior	2.74(0.65)

Table S2.

Descriptive statistics on economic and health context experimental task for healthy participants

	Economic context		Health Context	
Variables	M	SD	M	SD
Expected value				
M0	62.40	13.54	..	..
M1	61.68	13.64	113.67	28.32
M2	81.13	21.28	80.67	34.43
M3	60.98	11.71	102.16	27.33
Investment				
M0	27.76	7.24	..	..
M1	27.54	10.86	4.84	1.14
M2	31.17	10.42	5.33	1.01
M3	29.81	10.20	5.04	1.04
Feedback				
M0	91.29	17.57	..	..
M1	78.01	17.68	141.11	11.90
M2	119.26	26.55	73.80	29.44
M3	91.29	17.57	113.34	23.97

Table S3.

Repeated measure comparison (Friedman Non-parametric test) on economic and health related context experimental tasks for healthy participants

Variable	*Ecponomic Context (N=53)*				*Health Context (N=53)*			
	Friedman	*gl*	*p*	*W*	*Friedman*	*gl*	*p*	*W*
Expected Value	*54.62*	*3*	0.34	0.34	*41.00*	*2*	<0.001	0.39
Investment	*10.05*	*3*	*0.09*	*0.06*	*11.90*	*2*	*0.003*	*0.11*
Feedback	*85.93*	*3*	*<0.001*	*0.54*	*91.31*	*2*	*<0.001*	*0.86*

Variables	Economic context (N=53)	Healtth context (N=53)
	Sig.Aj	Sig.Aj
Expected Value		
M0-M1	1.000	--
M0-M2	<0.001***	--
M0_M3	1.000	--
M1-M2	<0.001***	<0.001***
M1-M3	1.000	0.098
M2-M3	<0.001***	<0.001***
Investment		
M0-M1	1.000	--
M0-M2	0.635	--
M0_M3	0.063	--
M1-M2	0.917	0.008**
M1-M3	0.107	0.522
M2-M3	1.000	0.296
Feedback		
M0-M1	1.000	--
M0-M2	<0.001***	--
M0_M3	<0.001***	--
M1-M2	<0.001***	<0.001***
M1-M3	<0.001***	<0.001***
M2-M3	0.002**	<0.001***

Table S4. (A)

Repeated measures comparison between each mediator for *Expected Value, Investment and Feedback* (Friedman Non-parametric test for main effects of mediator and posthoc tests) on economic and health related context experimental tasks for NoMC and MC groups

**NoMC**								
**Variable**	Economic Context (N=42)				Health Related Context (N=42)			
	Friedman	df	p	W	Friedman	df	p	W
**Expected Value**	12.86	3	**0.005**	0.11	17.71	2	**<0.001**	0.21
**Investment**	15.16	3	**0.001**	0.13	16.39	2	**<0.001**	0.19
**Feedback**	27.83	3	**<0.001**	0.24	47.81	2	**<0.001**	0.57

**MC**								
**Variable**	Economic Context (N=49)				Health Related Context (N=49)			
	Friedman	df	p	W	Friedman	df	p	W
**Expected Value**	16.91	3	**0.001**	0.12	16.10	2	**<0.001**	0.17
**Investment**	14.29	3	**0.003**	0.10	4.9	2	0.086	0.05
**Feedback**	48.82	3	**<0.001**	0.35	52.34	2	**<0.001**	0.56

Table S4. (B) Posthoc tests for each variable (expected, investment and feedback) on economic and health related context experimental tasks for NoMC and MC groups

	NoMC		MC		
	Economic context (N=42)	Health context (N=42)		Economic context (N=49)	Health context (N=49)
**Variables**	Sig.Aj	Sig.Aj	**Variables**	Sig.Aj	Sig.Aj
**Expected Value**			**Expected Value**		
M0-M1	1.000	--	M0-M1	1.000	--
M0-M2	0.136	--	M0-M2	**0.020**	--
M0-M3	1.000	--	M0-M3	1.000	--
M1-M2	**0.006**	**<0.001**	M1-M2	**0.040**	**<0.001**
M1-M3	0.107	**0.004**	M1-M3	1.000	0.061
M2-M3	1.000	1.000	M2-M3	0.093	0.365
**Investment**			**Investment**		
M0-M1	1.000	--	M0-M1	0.168	--
M0-M2	0.066	--	M0-M2	**0.021**	--
M0_M3	**0.010**	--	M0_M3	**0.005**	--
M1-M2	0.212	**0.001**	M1-M2	1.000	ND
M1-M3	0.039	1.000	M1-M3	1.000	ND
M2-M3	1.000	**0.023**	M2-M3	1.000	ND
**Feedback**			**Feedback**		
M0-M1	1.000	--	M0-M1	0.099	--
M0-M2	**<0.001**	--	M0-M2	**<0.001**	--
M0_M3	1.000	--	M0_M3	1.000	--
M1-M2	**<0.001**	**0.002**	M1-M2	**<0.001**	**<0.001**
M1-M3	1.000	**0.002**	M1-M3	1.000	**<0.001**
M2-M3	**<0.001**	**0.002**	M2-M3	**<0.001**	0.070
